# The effect of total anthocyanin-base standardized (*Cornus mas* L.) fruit extract on liver function, tumor necrosis factor α, malondealdehyde, and adiponectin in patients with non-alcoholic fatty liver: a study protocol for a double-blind randomized clinical trial

**DOI:** 10.1186/s12937-019-0465-z

**Published:** 2019-07-19

**Authors:** Zohreh Sadat Sangsefidi, Mahdieh Hosseinzadeh, Ali Mohammad Ranjbar, Mohsen Akhondi-Meybodi, Hossein Fallahzadeh, Hassan Mozaffari-Khosravi

**Affiliations:** 1 Nutrition and Food Security Research Center, Shahid Sadoughi University of Medical Sciences, Yazd, Iran; 2 Department of Nutrition, School of Public Health, Shahid Sadoughi University of Medical Sciences, Yazd, Iran; 3 Department of Pharmacognosy, Faculty of Pharmacy, Shahid Sadoughi University of Medical Sciences, Yazd, Iran; 4 Herbal Medicine Center, Faculty of Pharmacy, Shahid Sadoughi University of Medical Sciences, Yazd, Iran; 5 Gastroentrology Department, Shahid Sadoughi Hospital, Faculty of Medicine, Shahid Sadoughi University of Medical Sciences, Yazd, Iran; 6 Research Center of Prevention and Epidemiology of Non-Communicable Disease, Departments of biostatistics and Epidemiology, School of Public Health, Shahid Sadoughi University of Medical Sciences, Yazd, Iran

**Keywords:** Nonalcoholic fatty liver disease, Anthocyanins, Cornelian cherry, *Cornus mas* L

## Abstract

**Background:**

Nonalcoholic fatty liver disease (NAFLD) is one of the most common chronic liver diseases worldwide. Evidence showed that anthocyanins might have effects on NAFLD. Protective effects of Cornelian cherry (*Cornus mas* L.) extract, as an anthocyanins-rich source, on liver were reported in animal studies. However, very few clinical trials were conducted in this regard. Thus, the aim of this research will be to evaluate the effect of supplementation with total anthocyanin-base standardized cornelian cherry fruit extract on liver function (Serum levels of Alanine aminotransferase (ALT), Aspartate aminotransferase (AST), cytokeratin-18 fragment M30 (CK-18 M30), as well as steatosis and fibrosis of liver), tumor necrosis factor α (TNF-α), malondealdehyde (MDA), and adiponectin in patients with NAFLD.

**Methods:**

In a double-blind randomized clinical trial, 80 NAFLD patients will be studied. The patients will be randomly assigned into two groups. The intervention group will receive the cornelian cherry extract, containing 320 mg.d^− 1^ anthocyanins, per day for 12 weeks. The control group will also take the placebo daily for 12 weeks. Liver function (Serum levels of AST, ALT and CK-18 M30; steatosis and fibrosis of liver), serum levels of TNF-α, MDA, and adiponectin will be measured at the baseline and the end of trial for both groups and their results will be compared.

**Discussion:**

Considering evidences about the useful impacts of anthocyanins on NAFLD, the effects of supplementation with cornelian cherry extract will be investigated on the important variables related to NAFLD.

**Trial registration:**

Iranian Registry of Clinical Trials (IRCT20180419039359N1).

## Background

Nonalcoholic fatty liver disease (NAFLD) is one of the most prevalent chronic liver diseases [1–4]. The overall prevalence of NAFLD was from 5 to 20% among the healthy populations and more than 40% in the diabetic patients [5, 6]. However, a recent systematic review showed that its total prevalence was 33.9% among Iranian people [7]. The detailed pathogenesis and etiology of the disease are still unknown [8–10]. Nevertheless, NAFLD may be associated with factors such as insulin resistance [10, 11], oxidative stress, and adipokines such as adiponectin, cytokines, and other inflammatory mediators [10, 12]. Insulin resistance increases lipolysis in adipose tissue, releases free fatty acids to liver, and causes inflammation in liver [10, 11]. Moreover, mitochondrial dysfunction, oxidative stress, and increased inflammatory responses are related to damaged liver [10, 12]. The low level of serum adiponectin was also observed in NAFLD patients, which was related to the rate of steatosis, fibrosis, and severity of NAFLD [13]. Since no definite treatment has been found for this disease, identification of new therapeutic approaches is one of the current challenges [10, 14, 15]. A study suggested that flavonoids such as anthocyanins, found in plant sources with red, purple, and blue colors [16] were effective in treating NAFLD [17]. According to the literature, the effects of anthocyanins on NAFLD include decrease of lipid accumulation in the liver [18–23], improvement of insulin resistance [24–27], decrease of lipid profile [23–26], inflammation [19, 26], and oxidative stress [28–32]. Moreover, some human studies reported useful effects of anthocyanins on the levels of liver enzymes such as ALT and AST [33, 34], oxidative stress markers such as MDA [35], inflammatory markers such as TNF-α [36, 37], lipid profile [35, 36, 38–40], glycemic control [38, 39], insulin resistance [40, 41] and adiponectin [41]. However, very few clinical trials evaluated the effect of anthocyanins [37, 42, 43] on NAFLD. For example, supplementation with purified anthocyanins for 12 weeks was associated with a significant decrease in the level of Alanine Aminotransferase (ALT) and cytokeratin-18 fragment M30 (CK-18 M30). However, it improved the fibrosis scores of NAFLD patients [42]. In addition, another research reported that intake of *Hibiscus sabdariffa* extract, rich in anthocyanins, for 12 weeks improved liver steatosis in patients with fatty liver [43]. A study found that consumption of bayberry juice, as a source of anthocyanins, for 4 weeks decreased the levels of TNF-α and CK-18 M30 among NAFLD patients [37]. Nevertheless, the earlier studies had various limitations: lack of precise examination of steatosis and fibrosis of liver by an accurate none-invasive method such as transient elastography (Fibroscan) [37, 42, 43], low study power [42], lack of assessing other important factors such as adipokines, adiponectin, as well as inflammatory and oxidative stress markers [37, 42, 43]. Recent evidences demonstrated that results of transient elastography, as a non-invasive method, had appropriate consistency with results of biopsy, as an invasive but gold standard method for assessing NAFLD. Therefore, transient elastography can replace biopsy as a more accurate and none-invasive method in evaluating NAFLD [44–46]. *Cornus mas* L. (cornelian cherry) is a fruit rich in anthocyanins [47]. Although its protective effects on liver was reported in several animal studies [48–51], no clinical trial has ever investigated the effect of cornelian cherry fruit extract on NAFLD. Considering the high prevalence of NAFLD as well as the limited number and limitations of clinical trials over the effect of anthocyanins on NAFLD, further clinical trials are required in this area. The future trials should conduct comprehensive investigations on important NAFLD variables and examine the impacts of anthocyanins on NAFLD. Therefore, the present double-blind randomized clinical trial will be conducted to investigate the effect of supplementation with total anthocyanin-base standardized cornelian cherry fruit extract on the liver function (Serum levels of AST, ALT and CK-18 M30; steatosis and fibrosis of liver), TNF-α, MDA, and adiponectin in patients with NAFLD. Furthermore, serum concentrations of glucose, total cholesterol (TC), High density lipoprotein (HDL-C), low density lipoprotein (LDL-C), triglyceride (TG), insulin, and insulin resistance will be evaluated as the secondary outcomes.

## Methods

### Design and aim study

**Fig. 1:**
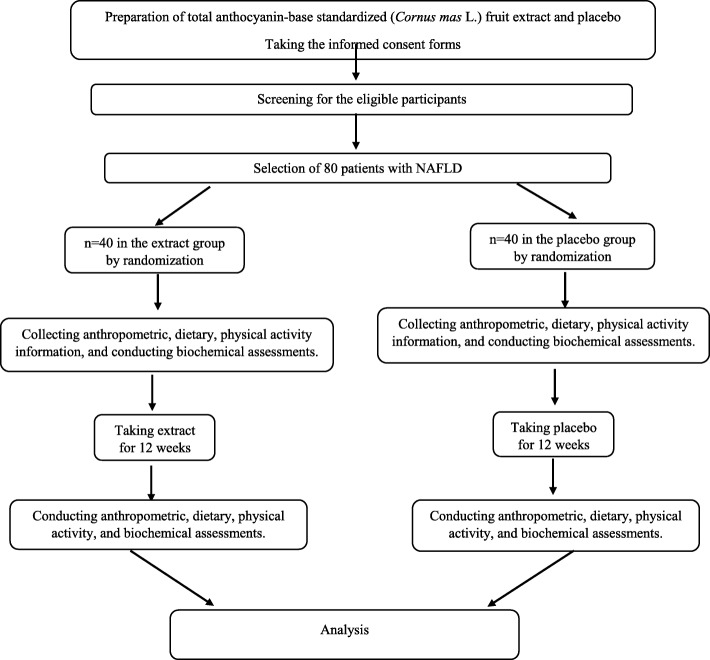
Protocol flow diagram. We will perform a double-blind randomized clinical trial to evaluate the effect of total anthocyanin-base standardized (*Cornus mas* L.) fruit extract on the liver function, tumor necrosis factor α, malondealdehyde, and adiponectin in patients with non-alcoholic fatty liver

This double-blind randomized parallel clinical trial will be carried out at the Department of nutrition, School of Public Health, Shahid Sadoughi University of Medical Sciences, Yazd, Iran. The main aim of this research is to investigate the effect of a 12-week supplementation with total anthocyanin-base standardized cornelian cherry fruit extract on liver function (Serum levels of AST, ALT and CK-18 M30; steatosis and fibrosis of liver), TNF-α, MDA, and adiponectin in patients with NAFLD. (Fig. 1 and Table 1). Moreover, serum concentrations of glucose, total TC, HDL-C, LDL-C, TG, insulin, and insulin resistance will be assessed as the secondary outcomes.

**Table 1:** Study timeline; we predicted 20 months to carry out the present study

Description of research activity	Duration (month)																							
	1	2	3	4	5	6	7	8	9	10	11	12	13	14	15	16	17	18	19	20	21	22	23	24
Reviewing the literature and writing the proposal	*																							
Preparing the total anthocyanin-base standardized (*Cornus mas* L.) fruit extract and the placebo		*	*	*																				
Standardizing the extract based on the total anthocyanin					*																			
Selecting the participants						*	*	*	*	*	*													
Collecting data, conducting the intervention and assessments												*	*	*										
Entering the data into SPSS and conducting the statistical analysis															*	*	*							
Providing the final reports																		*	*	*				

The symbol * represents the start and end time of each research activity in study timeline

### Preparation of total anthocyanin-base standardized cornelian cherry fruit extract

#### Materials and extraction

Fresh ripe berries of cornelian cherry were prepared from the forests of Ghazvin, Iran, in October 2018 and frozen at − 18 ∘C. Later, the fruits’ authenticity will be determined by specifying their voucher number (SSU0029) in the Department of Pharmacognosy, School of Pharmacy, Shahid Sadoughi University of Medical Sciences, Yazd. In the next stage, the fruits will be cleaned and their cores will be removed. Then, the fruit samples will be grinded and mixed with the solvents including ethanol 80% and HCL 0.1%. Extraction will be performed by percolation for one week. Then, it will be followed by Ultrasound-Assisted Extraction (UAE) for 20 min using an ultrasonic device (Elmasonic Easy 60 H, German; frequency 50/60 Hz). After filtration of the obtained extract, it will be concentrated by evaporation.

#### Total anthocyanin content determination

The total anthocyanin content of the achieved extract will be determined using the pH differential method [52, 53].

Since the prepared extract does not have purified anthocyanins, the extract’s total flavonoid/phenolic content should be measured. Furthermore, the necessary microbiology measurements will be performed for the prepared extract.

#### Preparation of placebo

The placebo will be prepared using diluted water, caramel color, allura red color, and natural flavorings with a color, appearance, taste, and texture similar to the cornelian cherry extract, but without any anthocyanins.

### Study population

Patients with NAFLD will be selected based on the inclusion and exclusion criteria from gastroenterology clinics affiliated to Diabetes Research Center and Shahid Sadoughi University of Medical Sciences, Yazd, Iran. The inclusion criteria will be having 25–65 years of age; ALT levels of higher than 30 U/L in men and higher than 19 U/L in women; diagnosis of the disease by a gastroenterologist; and grade 1, 2, and 3 fatty liver. Furthermore, patients should be residents of Yazd city and will be required to sign the written consent forms to participate in the research. In the current research, nonalcoholic steatohepatitis (NASH) will be defined based on the guidelines of American Gastroenterological Association and American Association for the Study of Liver Diseases [54]. According to this guideline, NASH is defined as the presence of hepatic steatosis proved by ultrasonography or inflammation with hepatocyte injury (ballooning) with or without fibrosis [54]. Moreover, NAFLD will be diagnosed by ultrasonography based on following criteria: an increase in hepatic echogenicity via renal echogenicity as a reference, presence of enhancement and lack of differentiation of periportal, and bile duct walls reinforcement due to great hyperechogenicity of the parenchyma [55]. Exclusion criteria will include: having any history of diseases such as cirrhosis, viral hepatitis, cardiovascular disease, diabetes, Wilson, and cancer; taking medications including corticosteroids, non-steroidal anti-inflammatory drugs, hypoglycemic agents, or any medicines that affect the blood glucose, tamoxifen, sodium valproate, methotrexate, amiodarone, anti-retroviral agents for HIV, probiotics; consuming any medicine or supplement that affect liver function as well as supplements with antioxidant and anti-inflammatory properties (such as vitamin D, vitamin E, omega-3, resveratrol) one month before the study; following a special diet one month before the study; being pregnant and breastfeeding; and consuming alcohol. We will also ask the patients to not consume the medicines in exclusion criteria list during the study. Moreover, data of the patients with any of the following conditions will not be analyzed at the end of study: patients who take less than 80% of the administered extract or placebo as well as participants who are under a special treatment due to a specific medical reason during the study.

### Ethical considerations and trial registration

This survey was approved by Ethics Committee of Shahid Sadoughi University of Medical Sciences, Yazd, Iran (approval number: IR.SSU.SPH.REC.1396.171). The research was also registered on Iranian Registry of Clinical Trials (IRCT registration code: IRCT20180419039359N1). After informing the participants about the study protocol, they will be asked to sign the informed consent forms.

### Sample size

To estimate the sample size, we considered 95% confidence interval; 80% power (α = 0.05 and β = 0.2); the changes of ALT levels in intervention group compared with the control group, and S = 24% to achieved the significance level of 16% in both study groups according to the study by Zhang et al. [42]. Therefore, the sample size was calculated as *n* = 36 for each group. Eventually, 40 patients will be recruited for each group considering the possible 10% loss.

### Randomization

Eligible patients will be assigned into two groups (*n* = 40 in each group) of the cornelian cherry fruit extract and the placebo group using the randomization method, which will be performed using the Random Allocation Software [56]. The participants will be also stratified based on age, gender, BMI, and severity of fatty liver (fatty liver grade 1, 2, 3).

### Intervention

The target daily dose of 320 320 mg.d^− 1^ of total anthocyanin will be administered for 12 weeks [42]; with the required daily intake of extract adjusted in accordance with the total anthocyanin content measured in the extract. Moreover, the total anthocyanin content in the extract will be measured in distinct intervals during the trial (on days 1, 6, 12, 20, and after 6 months). If it is required, we will adjust the amount of consumed extract based on its total anthocyanin content.

The control group will also receive the placebo, matched with the extract in terms of appearance, taste, color, and texture (but without any anthocyanins) for 12 weeks. The participants will be asked to keep the intervention and placebo, which are presented as liquid in the refrigerator to ensure food safety.

In the current double-blind randomized clinical trial, the cornelian cherry extract and the placebo will be packed in containers with the same color, shape, and size, so that they cannot be identifiable by the patients or administrators. A person out of research, who does not know about the details of study, will be asked to label the bottles containing the extract or placebo as A or B, the contents of these bottles should be consumed in one month by participants. This person will be asked to inform the researchers about his/her labeling selection at the end of study. We will also ask the participants to give back their bottles (empty or not) at the end of each month and receive the next bottles until the end of study. At the end of intervention, the remaining contents of bottles will be recorded for each participant. Moreover, regular consumption of the extract or placebo will be checked by contacting the patients during the survey. At the end of the trial, the consumed amounts of extract or placebo will be estimated considering the remaining contents of the bottles for each participant. In the case that a patient in each group consumed less than 80% of the administered extract or placebo, his/her data will not be analyzed at the end of the study.

### Data collection

Data on patients’ demographic information, history of diseases, and consumption of medications or supplementations will be collected using a general questionnaire at the beginning of the research.

Participants’ dietary intakes will be evaluated using a three-day food record (2 week days and 1 weekend day) at the baseline and at the end of trial. Then, to estimate the total energy and macronutrients’ intakes, Nut IV (the Hearst Corporation, San Bruno, CA) will be used. Moreover, in order to evaluate the patients’ physical activity levels, the short form of International Physical Activity Questionnaire (IPAQ) will be administered at the beginning and the end of survey. Meanwhile, anthropometric parameters (e.g., weight, height, Body Mass Index (BMI), and waist and hip circumference) will be measured with minimal clothing and without shoes at the baseline and at the end of study. Body weight will be assessed by a seca scale with an accuracy of 100 g. Height will be measured in a relaxed position with the accuracy of 0.5 cm by a seca stadiometer. Then, BMI will be calculated after dividing the body weight (kg) by the square of height (m). Waist circumference will be also measured at the midway between the lowest ribs and the iliac crest. Moreover, hip circumference will be measured over the largest part of the buttocks.

### Outcomes measurements

At the baseline and at the end of the research, 10 ml of blood sample will be taken from each participant after 10–12 h of overnight fasting for biochemical analyses.

#### Primary outcomes

At the baseline and at the end of trial, serum concentrations of Alanine aminotransferase (ALT) and Aspartate aminotransferase (AST) will be measured with an auto-analyzer. Serum levels of TNF-α, MDA, adiponectin, and CK-18 M30 will be also determined by Enzyme-linked immunosorbent assay (ELISA).

Furthermore, hepatic steatosis and liver fibrosis will be examined using transient elastography by a gastroenterologist at the baseline and at the end of the survey. Evaluation will be conducted while patients are lying in a dorsal decubitus position with their right arm in maximum abduction [57].

#### Secondary outcomes

At the baseline and at the end of the study, serum concentrations of glucose, total cholesterol (TC), high density lipoprotein (HDL-C), low density lipoprotein (LDL-C), and triglyceride (TG) will be determined by an auto-analyzer. Serum levels of insulin will be also measured by ELISA.

Moreover, insulin resistance will be evaluated at the baseline and at the end of trial by the following two methods:

- Homeostasis model assessment – insulin resistance (HOMA-IR) = fasting insulin (μU/ml) × fasting glucose (mg/dl)/405.
- Quantitative insulin sensitivity check index (QUICKI) = 1/log fasting insulin (μU/ml) + log fasting glucose (mg/dl).

### Statistical analysis

Data analysis will be conducted using SPSS version 24 (SPSS Inc., Chicago, IL, USA). The normality of variables will be evaluated using Kolmogorov- Smirnov test. Qualitative and quantitative variables will be presented as numbers (percentages) and mean ± Standard Deviation (SD) or medians with interquartile ranges, respectively. Then, the Chi-Squared test will be used to compare the qualitative variables between two groups. In addition, the variables with normal distribution will be compared between and within the groups using the independent sample t-test and the paired sample t-test, respectively. However, in order to compare the variables with non-normal distribution between and within the groups, Mann-Whitney test and Wilcoxon test will be applied, respectively. To control the confounding variables, analysis of covariance (ANCOVA) will be used to examine the differences in post-intervention values between the two groups while adjusting for the baseline values and covariates. A *p*-value of < 0.05 will be considered as the statistically significant level.

## Discussion

Nonalcoholic fatty liver disease is one of the most common chronic liver diseases with an increasing prevalence throughout the world. Since no certain therapy has been found for NAFLD yet, determination of new and effective treatments is one of the substantial challenges of the current researchers. Recently, studies indicated that anthocyanins had some impacts on NAFLD. In addition, the protective effects of cornelian cherry fruit extract, as an anthocyanins-rich source, on liver were reported in animal studies. However, very few clinical trials investigated the impact of anthocyanins on NAFLD and we are faced with paucity of information on the disease and its main limitations. Therefore, further clinical trials with a more comprehensive assessment of important variables related to NAFLD are needed to evaluate the impact of anthocyanins on patients with NAFLD. Therefore, we will examine the effect of supplementation with total anthocyanin-base standardized cornelian cherry fruit extract on liver function, TNF-α, MDA, and adiponectin in NAFLD patients. The findings of this study can provide new information over the impact of total anthocyanin-base standardized cornelian cherry fruit extract, as an anthocyanins-rich source, on NAFLD. It also paves the way to discover new approaches for treatment of the disease.

## Data Availability

Not applicable.

## Abbreviations

ALT: Alanine aminotransferase
AST: Aspartate aminotransferase
BMI: Body Mass Index
CK-18 M30: Cytokeratin-18 fragment M30
ELISA: Enzyme-linked immunosorbent assay
HDL-C: High density lipoprotein
HOMA-IR: Homeostasis model assessment – insulin resistance
IPAQ: International Physical Activity Questionnaire
LDL-C: Low density lipoprotein
MDA: Malondealdehyde
NAFLD: Nonalcoholic fatty liver disease
QUICKI: Quantitative insulin sensitivity check index
SD: Standard Deviation
TC: Total cholesterol
TG: Triglyceride
TNF-α: Tumor necrosis factor α
